# Oral language skills, callous and unemotional traits and high-risk patterns of youth offending

**DOI:** 10.1007/s00787-022-01980-1

**Published:** 2022-04-11

**Authors:** Stavroola A. S. Anderson, David J. Hawes, Pamela C. Snow

**Affiliations:** 1 School of Psychology, The University of Sydney, Sydney, NSW Australia; 2 School of Education, La Trobe University, Bendigo, VIC Australia

**Keywords:** Youth offenders, Callous-unemotional traits, Oral language, Violence, Age of onset

## Abstract

Extensive research has associated adolescent delinquent behavior with verbal deficits, yet for some subgroups of youth offenders better verbal ability has been associated with increased risk. This study examined associations between specific oral language skills and established markers of high-risk youth offending comprising callous and unemotional (CU) traits, early age of the first offence, and violent offending. Measures of language, CU traits, anxiety, as well as official youth justice data, were collected for adolescent male offenders and non-offenders (*n* = 130; aged 13–19 years; 62% youth offenders). Pragmatic language was found to be differentially associated with distinct variants of CU traits based on high/low levels of anxiety. Furthermore, among youth offenders with primary variant (low anxiety) CU traits, more violent offending was associated with better structural language skills, while earlier age of first offence was associated with better pragmatic language skills.

## Introduction

Extensive research has associated antisocial behavior with risk factors related to verbal ability. Low verbal ability has not only been associated with an increased risk of offending [61], but an increased risk of violent offending [37], and an earlier onset of offending [42]. This is noteworthy, as an early age of onset is characteristic of a particular chronic and severe trajectory of antisocial behavior [34], as reflected in Moffitt’s influential taxonomy [41]. This model distinguishes between a relatively low-risk trajectory of antisocial behavior that commences and desists during adolescence, and a particularly high-risk life-course-persistent trajectory that is often initiated early in childhood and involves greater violence. Current diagnostic criteria further subdivide youth with early-onset conduct disorder into those with or without limited prosocial emotions, more generally referred to as callous-unemotional (CU) or psychopathic traits (e.g., lack of guilt and empathy) [16]. Importantly, research into CU traits has also informed emerging accounts of language and offending, as follows.

In early research, Loney et al. [35] found that antisocial youth with low CU traits demonstrated a deficit in verbal ability, while those with high CU traits did not. Munoz et al. [43] subsequently found higher verbal ability to be associated with lower levels of violent offending among youth low in CU traits, but with higher levels of violent offending for youth high in CU traits. Other research, however, has found no evidence that CU traits moderate the relationship between verbal ability and antisocial behavior in adolescent offenders [3].

Potential explanations for these mixed findings can be found in emerging research into distinct variants of CU traits based on co-occurring levels of anxiety (i.e., a low anxiety primary variant, and a high anxiety secondary variant) [28]. This distinction is potentially important given the distinct developmental and neurocognitive deficits associated with each. Individuals with primary variant CU traits tend to be relatively free from childhood maltreatment [9], score low on measures of psychological distress [6], and demonstrate less engagement with distressing emotional stimuli [28]. In contrast, secondary variant CU traits are associated with more severe childhood maltreatment [6], greater emotional and attentional problems [28], and more severe externalizing behavior [25]. Accordingly, the antisocial outcomes of individuals with secondary variant CU traits are assumed to be accounted for largely by adverse environmental influences, whereas those of individuals with primary variant CU traits are thought to implicate more neurodevelopmental underpinnings [28].

Importantly, previous research has relied largely on global measures of verbal ability that may mask potentially important individual differences in oral language skills. Oral language is understood to consist of five key domains [20]. Four of these domains: phonology, morphology, syntax (all representing aspects of language form), and semantics (language content), involve rules that relate sound combinations to meaning, and are together generally categorized as structural language [46]. While structural language continues to develop throughout childhood, key elements are typically established during early childhood [20]. The fifth domain of oral language, pragmatics, encompasses the subtleties of the appropriate use of language in social situations. It is interconnected with the development of a range of other socio-cognitive skills, and continues to develop beyond childhood and adolescence [57]. Importantly, as expectations and skills related to social cognition increase during adolescence, there is typically a marked increase in the complexity of pragmatic language skills associated with this developmental period [45]. Pragmatic skills are diverse, yet often emphasize the use of contextual cues to infer meaning [4].

Antisocial behavior during adolescence, including criminal offending, has been associated with deficits in structural [21] and pragmatic [44] language skills. However, relatively little research has investigated associations between these specific oral language skills and specific forms of antisocial behavior, including subtypes defined by the level of CU traits. Evidence suggests that individuals with high levels of psychopathic traits may possess structural language skills, such as those related to phonological processing and semantics, that are comparable [11], or potentially superior to [53], those low in psychopathic traits. However, findings indicate that high-psychopathy individuals may have deficits in the subtler skills associated with pragmatic language. Adolescent [51] and adult offenders [19] with high levels of psychopathic traits have been shown to exhibit weaker or inefficient patterns of language lateralization, suggesting that they may have different cognitive resources available during language-rich tasks. Evidence further suggests that high-psychopathy individuals may have deficits in specific pragmatic language skills, compared to low-psychopathy individuals. In adult offenders, high levels of psychopathy have been associated with poor pragmatic language, such as difficulty accurately interpreting metaphors with emotional content [18], and categorizing words in abstract tasks [27]. Further, adolescent offenders high on psychopathy demonstrate poorer performance on oral and written comprehension tasks [17, 59], suggesting difficulties making inferences from language-based information sources.

In summary, antisocial youth low in CU traits appear to exhibit poor oral language skills compared to antisocial youth high in CU traits, or typically developing youth. However, among high CU youth, individuals with primary versus secondary variants of CU traits appear to follow risk pathways that implicate distinct oral language skills. High verbal ability has been associated with an earlier age of first offence among psychopathic adults [23] and with more violent offending among youth with high CU traits [43]. Importantly, research has yet to examine whether superior oral language skills are associated with an earlier age of onset, or more violent offending, among individuals with primary variant CU traits in particular. This would seem likely given the apparent neurodevelopmental underpinnings of primary variant CU traits.

The aim of the current study was to examine patterns of antisocial offending and oral language skills among adolescents with primary versus secondary variants of CU traits. The first specific aim was to examine the oral language skills that characterize adolescents with primary versus secondary variants of CU traits. It was hypothesized, first, that associations between CU traits and particular language skills would vary based on variant of CU traits. Specifically, it was predicted that higher CU traits would be associated exclusively with poorer pragmatic language skills, but only among youth with primary variant CU traits, while higher CU traits would be associated with both poorer structural and pragmatic language skills among youth with secondary variant CU traits. The second specific aim was to examine the interaction between variants of CU traits, and language skill, in relation to patterns of youth offending. The second hypothesis was that offenders with primary variant CU traits and better language skills would exhibit earlier onset of offending, and more violent offending, than those with secondary variant or low CU traits, across both structural and pragmatic language skills.

## Methods

### Participants

The sample consisted of 130 male adolescents between the ages of 13 and 19 years (*M* = 16.32, *SD* = 1.35), residing in New South Wales (NSW), the most populous state in Australia. With regard to inclusion criteria, participants were eligible for the study if they had undertaken the majority of their schooling in an English-speaking country, had no known diagnosis of intellectual disability or hearing impairment, and were not known to be experiencing an acute episode of mental illness. Most participants reported being of non-Indigenous Australian ethnicity (53.8%; majority Caucasian), while a substantial proportion reported being of Indigenous ethnicity (46.2%; majority Aboriginal). All participants reported that their primary language of communication was Standard Australian English. Socio-economic status (SES) was calculated through the assignment of an Index of Relative Socio-Economic Advantage/Disadvantage (IRSAD; from one (lowest) to nine (highest)), based on the postcode of usual residence [2]. Participants had a mean IRSAD of 3.26, indicating a relatively high disadvantage and a lack of advantage in general. Based on self-reported years of schooling completed, participants had experienced an average school education of between 9 and 10 years (*M* = 9.56, *SD* = 1.53).

Eighty-one participants were youth offenders, recruited through youth justice agencies throughout NSW. Forty-nine participants were non-offenders, recruited through public secondary schools throughout NSW. Schools were selected to maximise the potential for matching ethnicity and SES with the youth offender group (based on information provided by the Australian Bureau of Statistics and Department of Education, NSW). Preliminary analyses revealed no significant differences between the youth offender and non-offender groups on ethnicity, primary language of communication, or years of schooling. A significant difference between groups was found for SES, however, it was the non-offender group which demonstrated a lower mean SES than the youth offender group. Due to the different age cohorts under the jurisdiction of public secondary schools (13–18 years) and youth justice agencies (14–21 years) in NSW, there was a significant age difference between groups. However, while the non-offender group had a younger average age, there was no significant difference between the two groups in terms of total years of schooling. In attempting to match youth offender with non-offender samples across a number of demographic variables, previous researchers have similarly involved non-offender groups of a younger age, but with equivalent education to the youth offender group [e.g., 21, 58].

### Measures

CU traits were measured using the Inventory of Callous-Unemotional Traits (ICU), a self-report scale consisting of 24 items each rated on a four-point scale [15]. A higher total score indicates higher levels of callous-unemotional traits. The reliability and validity of the scale have been supported in research involving youth offenders [49]. In the current study, the internal consistency of the total scale was high (Cronbach’s *α* = 0.80).

Anxiety was measured using the Anxious-Depressed subscale of the Youth Self Report (YSR; part of the Achenbach System of Empirically Based Assessment) [1]. The YSR is a widely used questionnaire designed to assess psychosocial functioning in adolescents. It has been normed for ages 11–18 years and has been shown to have sound reliability and validity in samples of youth offenders [30]. The Anxious-Depressed subscale consists of 16 items that are scored on a three-point scale, and versions of the subscale have been used to delineate primary and secondary variants of CU traits in a number of previous studies [e.g., 12, 25, 29]. The internal consistency of the subscale was high in the current study (Cronbach’s *α* = 0.81).

Structural language was assessed with the Clinical Evaluation of Language Fundamentals, Fourth Edition, Australian Standardization (CELF4-A) [54]. Versions of the CELF have been widely used in international research investigating the oral language skills of youth offenders [31]. The CELF4-A was normed on a representative Australian sample and has standard scores for ages 5:0–21:11 years. Four subtests of the CELF4-A were administered: Recalling Sentences; Formulated Sentences; Word Classes; and Word Definitions. Raw scores for each subtest were converted to standard scores, which were then summed to derive a Core Language Score.

Pragmatic language was measured using the Social Inference-Minimal Task (SI-M) of The Awareness of Social Inference Test (TASIT) [39]. TASIT was designed to differentiate between neurologically typical and neurologically compromised individuals aged 13–60 years. In the SI-M Task, participants view a series of 15 short videotaped vignettes of actors interacting in conversational exchanges. Five of these scenes represent sincere exchanges, where words and meaning are consistent, and 10 represent sarcastic exchanges, in which paralinguistic cues (e.g., tone of voice) indicate an inconsistency between words and meaning. After watching each scene, participants were asked four questions and were allocated a total of up to four points for each scene. Points were then summed to produce a total score.

Criminal offending was operationalized as a categorical variable with two levels (youth offender; non-offender). Categorization as a youth offender was based on officially documented contact with a youth justice agency. Categorization as a non-offender was based on a self-reported lack of official contact with a youth justice agency. Officially recorded history of offending for participants in the youth offender group was provided by the youth justice agency. Official first contact with youth justice was used to measure the age of first offence. Official number of violent offences was used to measure violent offending. Violent offences were classified based on Australian and New Zealand Standard Offence Classification divisions and codes [50].

### Procedure

This research was approved by the University of Sydney Human Research Ethics Committee, as well as the NSW Department of Communities and Justice, and the NSW Department of Education. To avoid the perception of coercion, initial contact with potential participants was made through youth justice staff (for youth offenders) and education staff (for non-offenders). These staffs were bound by legislation and codes of conduct concerning interactions with young people in their care. The researcher distributed inclusion criteria, the participant information and consent forms to participating youth justice centers and schools. To broadly match the offender and non-offender samples on demographic variables, staff in schools were requested to approach students who had low SES and/or Indigenous backgrounds, and a range of academic and behavioural capabilities. The researcher then visited each participant for the purpose of onsite data collection. Data collection occurred between October 2014 and May 2016.

The researcher made it clear to participants at the beginning of each assessment session that there were no consequences for non-participation or withdrawal from the research or their responses to the language tasks or self-report questions. The researcher demonstrated to participants that only an alpha-numeric code, not their name or other identifying information, was recorded on each data collection form. The researcher explained that the spreadsheet containing participant names would be accessible only to a very limited number of researchers, and not the staff who worked with them directly. Testing sessions were conducted in a space familiar to the participant, and in which they were likely to feel safe. All participants were easily able to access a youth justice or education staff member if they wished to do so during these sessions. It was made clear to participants prior to commencement of testing that the researcher was obliged to inform youth justice or education staff if the participant disclosed any information that led to concern for their health or safety. However, the researcher assured participants that specific responses to items in measures would not be shared with staff.

Testing commenced with a semi-structured interview followed by an assessment of nonverbal ability with the Matrices subtest of the Kaufman Brief Intelligence Test, 2nd edition (KBIT-2) [26]. The CELF4-A, TASIT-SI-M, ICU and YSR were then presented to participants in random order. Random sequencing of measure presentation was utilized to minimize the potential impact of order effects [56], which have been noted in research involving multiple measures of related aspects of cognition (such as oral language skills) [33]. To account for potentially low literacy levels, all items on the CELF4-A and TASIT-SI-M were read to participants. Consistent with the respective test manuals, demonstrations and practice opportunities for each subtest were provided. The researcher gave participants the option of having items on the ICU and YSR read to them, and their responses recorded for them.

### Data analytic plan

All analyses were conducted using SPSS, Version 24 [22]. An a priori power analysis was conducted using G*Power 3.1 [14], and recommendations by Dattalo [10]. Based on the assumptions of an alpha of 0.05, a power of 0.95, and a medium effect size (*Cohens f*^*2*^ = 0.15), it was determined that the minimum desired sample size was 76.

Hypothesis one was tested using two hierarchical regression models, with a separate model for each of the structural and pragmatic language outcome variables. To identify whether associations with language skills were independent of demographic factors, two centered continuous variables (age, SES), and two recoded weighted categorical variables (ethnicity, offender status) were included in each model. In each model, demographic variables (age, SES, ethnicity, offender status), as well as CU traits and anxiety, were entered in step one, and the interaction term (CU x anxiety), was entered in step two. Preliminary analyses revealed no violation of the assumptions of normality, linearity, multicollinearity, or homoscedasticity.

Hypothesis two was tested within the youth offender subsample (*n* = 81), through a separate multiple analysis of covariance (MANCOVA) for each of the two language variables. To maximize the size of non-overlapping groups, a two-step process was used to create three groups. First, to distinguish between individuals with high versus low levels of CU traits, a median split on the youth offender group ICU Total Score was used to form a low CU (group one; *n* = 37) and a high CU group. Second, to distinguish variants within the high CU group, a median split on the high CU group’s YSR Anxious-Depressed subscale score was used to form secondary variant CU traits (high anxiety; group two; *n* = 23) and primary variant CU traits (low anxiety; group three; *n* = 21) groups. This approach to testing the moderating role of CU traits/variants using relatively small subsample groups is consistent with methodological recommendations [10], and prior research [43, 55].

To test for CU variant as a moderator of the association between language skills and high-risk offending, high and low groups were formed for each language variable. This was done by performing median splits on youth offenders’ scores for structural (high: *n* = 41; low: *n* = 40) and pragmatic (high: *n* = 37; low: *n* = 44) language. To ensure that associations with patterns of offending were independent of demographic factors, age, SES, and ethnicity were included as covariates in each analysis. The two dependent variables used to examine high-risk patterns of offending were age of first offence and violent offending. Preliminary analyses revealed that scores for violent offending were positively skewed and contained a number of outliers and zero values. Therefore, all MANCOVA were repeated with this variable replaced by an alternative value based on log-transformed scores, which corrected for any violations of statistical assumptions. However, given that results did not differ between the two sets of analyses, the values for the non-transformed data are reported here. Checks revealed no further violations of assumptions of normality, linearity, homogeneity of variances, or homogeneity of regression slopes.

## Results

### Descriptive statistics

Means, standard deviations and zero-order correlations for relevant study variables for the whole sample and the youth offender subsample are shown in Table 1. For the whole sample (*n* = 130), higher levels of CU traits were associated with poorer structural language skills. In addition, status as a youth offender was associated with higher levels of CU traits and anxiety, and poorer structural and pragmatic language skills. Within the youth offender subsample (*n* = 81), higher levels of CU traits were associated with an earlier age of first offence, while higher levels of anxiety were associated with a later age of first offence. In addition, superior pragmatic language skills were associated with a later age of first offence. As shown in Table 1, lower levels of education were significantly correlated with higher levels of CU traits, and with anxiety among youth offenders. Education demonstrated no significant association with either structural or pragmatic language. Higher nonverbal ability was significantly correlated with better structural and pragmatic language skills but demonstrated no association with either CU traits or anxiety.

**Table 1:** Descriptive Statistics and Zero Order Correlations for whole sample (top) and youth offender subsample (bottom)

Whole sample (*n* = 130)											
	*M*	*SD*	Correlation								
			1	2	3	4	5	6	7	8	9
1. CU traits	26.08	8.21									
2. Anxiety	5.65	4.21	−0.09								
3. Structural language	79.55	20.84	−0.18*	0.01							
4. Pragmatic Language	48.41	6.91	−0.01	0.02	0.57***						
5. Age	16.38	1.36	0.13	0.18*	−0.36***	0.05					
6. Ethnicity	1.54	0.5	−0.11	0.03	0.30***	0.22*	−0.09				
7. SES	3.26	1.87	0.08	0.002	0.08	0.20*	0.06	0.03			
8. Education	9.56	1.53	−0.23**	0.17	0.01	0.14	0.47***	0.03	−0.08		
9. Non-verbal ability	92.4	13.67	−0.05	0.08	0.64***	0.44***	−0.12	0.09	0.1	0.12	
10. Offender status	1.38	0.49	−0.26***	−0.18*	0.59***	0.32^***^	−0.48***	0.08	−0.19*	0.07	0.43***

Youth offenders (*n* = 81)												
	*M*	*SD*	Correlation									
			1	2	3	4	5	6	7	8	9	10
1. CU traits	27.74	8.62										
2. Anxiety	6.23	4.18	−0.2									
3. Structural language	70	17.17	0.02	0.1								
4. Pragmatic language	46.68	7.3	0.14	0.11	0.56***							
5. Age	16.88	1.28	0.01	0.13	−0.15	0.19						
6. Ethnicity	1.51	0.5	−0.1	−0.01	0.25*	0.16	−0.03					
7. SES	3.54	2.12	0.14	−0.08	0.2	0.30**	−0.04	−0.04				
8. Education	9.48	1.74	−0.29**	0.26*	−0.05	0.07	0.47***	0.09	−0.05			
9. Non-verbal ability	87.89	11.41	0.17	0.12	0.46***	0.38***	0.02	−0.08	0.23*	−0.01		
10. Age first offence	14.83	1.67	−0.25*	0.29**	0.00	0.23*	0.51***	0.14	0.04	0.57***	0.03	
11. Violent offending	3.1	3.66	0.12	0.07	0.07	0.05	0.21	−0.13	0.18	−0.16	0.13	−0.17

*Variable* CU Traits (ICU total scale score); Anxiety (YSR anxious-depressed subscale score); Structural Language (CELF-4, Core Language Score); Pragmatic Language (TASIT, Social Inference Minimal, sum of scores); Age (years); Ethnicity (Indigenous = 1, Non-Indigenous = 2); SES (IRSAD categories); Education (total years schooling completed); Nonverbal Ability (KBIT-2 Matrices subtest standard score); Offender Status (Youth Offender = 1, Non-Offender = 2); Age First Offence (years, youth justice data); Violent Offending (total violent offences, youth justice data)

***p ≤ 0.001; **p ≤ 0.01; *p ≤ 0.05

### Tests of main study hypotheses

Coefficients for the two hierarchical regression models testing predictors of structural and pragmatic language are provided in Table 2. In the regression model testing predictors of structural language there was no significant main effects for CU traits or anxiety, and no significant interaction between CU traits and anxiety. In the model testing predictors of pragmatic language there was no significant main effects for CU traits or anxiety. There was a significant interaction between CU traits and anxiety. This interaction was probed by testing the conditional effects of CU traits on pragmatic language at high and low anxiety. CU traits were negatively related to pragmatic language when anxiety was high (*ß* = -0.25, *p* = 0.024), but positively when anxiety was low (*ß* = 0.41, *p* < 0.001). That is, higher scores on CU traits were associated with poorer pragmatic language for youth high in anxiety (i.e., secondary variant CU traits), but with better pragmatic language for youth low in anxiety (i.e. primary variant CU traits).

**Table 2:** Hierarchical Regression Analyses for Structural and Pragmatic Language Skills with predictors CU Traits and Anxiety

	Structural language			Pragmatic language		
	*β*	*b *[95% CI]	*R*^2^	*β*	*b *[95% CI]	*R*^2^
Age	−0.09	−1.43 [−3.72, 0.86]		0.28**	1.42 [0.54, 2.30]	
Ethnicity	0.23***	9.72 [4.23, 15.22]		0.20*	2.80 [0.69, 4.91]	
SES	0.19**	2.06 [0.57, 3.54]		0.27***	0.99 [0.42, 1.56]	
Offender status	−0.59***	−25.25 [−31.97, −18.53]		−0.53***	−7.52 [−10.10, −4.94]	
ICU	0.01	0.04 [−0.31, 0.38]		0.1	0.08 [−0.05, 0.22]	
Anxiety	0.12	0.61 [−0.05, 1.28]	0.47***	0.06	0.10 [−0.16, 0.36]	0.28***
ICU × anxiety	−0.12	−0.07 [−0.15, 0.01]	0.48	−0.33***	−0.07 [−0.10, −0.04]	0.38***

*Variables*. Age (centered); Ethnicity (dichotomized with weighted effect size: Indigenous Australian = −0.54; non-Indigenous Australian = 0.46); SES (centered); Offender Status (dichotomized with weighted effect coding: Youth offender = 0.38; Non-offender = −0.62); CU Traits (ICU total scale score; centered); Anxiety (YSR anxious-depressed sub-scale score; centered); Language: Structural (CELF-4, Core Language Score; centered); Pragmatic (TASIT, Social Inference Minimal, sum of scores; centered)

***p ≤ .001; **p ≤ .01; *p ≤ .05

*β* standardized beta, *b* unstandardized beta, *CI* confidence interval

Statistics for the two MANCOVA conducted for structural and pragmatic language skills can be seen in Table 3. In each of these analyses, associations were examined between independent variables, comprising the respective language skill (high/low), CU traits variant (primary/secondary), and the dependent variables of age of first offence and violent offending. In the analyses examining structural language, no main effects were found. A significant interaction was found between CU variant and structural language, which univariate analysis revealed was only significant for violent offending. Pairwise comparisons using Bonferroni adjustments revealed a significant difference in violent offending for primary variant CU youth (*M*_*DIFF*_ = − 4.72, *SE* = 1.56, *p* = 0.003). Youth with stronger language skills demonstrated significantly more violent offending (*EMM* = 5.19, *SE* = 1.04) than those with poorer language skills (*EMM* = 0.47, *SE* = 1.14). In the analyses examining pragmatic language, no significant main effects were found. A significant interaction was found between CU variant and pragmatic language, which univariate analyses revealed was significant for age of first offence only. Pairwise comparisons with Bonferroni adjustments revealed a significant difference in age of first offence for primary variant CU youth (*M*_*DIFF*_ = 1.44, *SE* = 0.66, *p* = 0.032) and low CU youth (*M*_*DIFF*_ = − 1.15, *SE* = 0.51, *p* = 0.025). For youth with primary variant CU traits, a significantly earlier age of first offence was demonstrated by those with better language skills (*EMM* = 14.12, *SE* = 0.34), compared to those with poorer language skills (*EMM* = 15.56, *SE* = 0.55). For youth with low CU traits, a significantly earlier age of first offence was demonstrated by those with poorer language skills (*EMM* = 14.81, *SE* = 0.28), compared to those with better language skills (*EMM* = 15.97, *SE* = 0.41).

**Table 3:** Interactive Effects of Variant of Callous-Unemotional Traits and Oral Language Skill on Age of First Offence and Violent Offending

Structural	Callous-unemotional variant								Part
	Low language skill			High language skill			*V*^(s)^	*F*	*η*^2^
	Primary CU (*n* = 9)	Secondary CU (*n* = 10)	Low CU (*n* = 21)	Primary CU (*n* = 12)	Secondary CU (*n* = 13)	Low CU (*n* = 16)			
Offending							0.13	2.56*	0.07
Age 1st offence	14.85 (0.48) [13.90, 15.80]	14.25 (0.45) [13.53, 15.32]	14.87 (0.33) [14.22, 15.51]	14.33 (0.44) [13.46, 15.19]	14.58 (0.40) [13.79, 15.37]	15.60 (0.36) [14.88, 16.31]		1.23	0.03
Violent	0.47 (1.14) −1.81, 2.75]	4.61 (1.08) [2.46, 6.77]	2.17 (0.78) [0.62, 3.72]	5.19 (1.04) [3.12, 7.27]	3.16 (0.95) [1.27, 5.05]	3.23 (0.86) [1.52, 4.94]		4.33*	0.11

Pragmatic	Primary CU (*n* = 6)	Secondary CU (*n* = 13)	Low CU (*n* = 25)	Primary CU (*n* = 15)	Secondary CU (*n* = 10)	Low CU (*n* = 12)			
Offending							0.15	2.94*	0.08
Age 1st offence	15.56 (0.55) [14.46, 16.66]	14.77 (0.38) [14.02, 15.52]	14.81 (0.28) [14.26, 15.37]	14.12 (0.36) [13.41, 14.84]	14.19 (0.43) [13.33, 15.05]	15.97 (0.41) [15.16, 16.78]		5.58**	0.13
Violent	0.84 (1.46) [−2.08, 3.76]	3.82 (0.99) [1.84, 5.80]	2.95 (0.74) [1.47, 4.42]	3.91 (0.95) [2.02, 5.80]	3.80 (1.14) [1.53, 6.07]	2.17 (1.07) [0.03, 4.31]		1.62	0.04

Reported results are estimated marginal means, standard errors (in parentheses) and 95% confidence intervals [in square brackets]; Multivariate tests: *V*^*(s)*^ = Pillai’s trace; *df* = 4, 144; ***p ≤ .001; **p ≤ .01; *p ≤ .05. Univariate tests: *df* = 2, 72; ***p ≤ .001; **p ≤ .01; *p ≤ .025 (Bonferroni adjustment). Co-variates were evaluated at: Age = 16.88; Ethnicity = 1.51; SES = 3.54. *Variables*. CU Variant—Primary: high CU traits, low anxiety; Secondary: high CU traits, high anxiety; Low: low CU traits (based on ICU total score, YSR anxious-depressed sub-scale score); Language: Structural (CELF-4, Core Language Score); Pragmatic (TASIT, Social Inference Minimal, sum of scores)

Additional post-hoc checks confirmed that the results of the multivariate analyses also remained significant when nonverbal ability and education were included in the models.

## Discussion

The current study examined associations between patterns of youth offending, specific oral language skills, and primary/secondary variants of CU traits. Descriptive analyses revealed that mean scores for the key variables of CU traits [29], anxiety [30], structural language [58] and pragmatic language [38] were consistent with prior research. In addition, bivariate associations between key variables demonstrated an expected pattern in which status as a youth offender was associated with higher scores for CU traits and anxiety, but lower scores for structural and pragmatic language. While higher scores for CU traits were found to be associated with an earlier age of first offence for youth offenders, no association was found between levels of CU traits and the extent of violent offending.

Findings from multivariate analyses supported the notion that associations between CU traits and oral language skills vary as a function of CU traits variant. That is, this association was moderated by anxiety. As predicted, the interaction between CU traits and anxiety was not uniform but varied depending on language skill. Specifically, the interaction was seen for pragmatic language, with higher CU traits associated with poorer pragmatic language skills, but only among youth with secondary variant CU traits (i.e., high CU traits and high anxiety). This is consistent with previous findings that high-psychopathy individuals perform poorly on pragmatic language tasks [27, 59]. These findings represent the first evidence, to our knowledge, that language-related correlates of CU traits among youth offenders differ according to variant of CU traits.

Contrary to predictions, higher CU traits were associated with superior skills in pragmatic language among youth with primary variant CU traits. This association was found to be independent of other participant characteristics, including antisocial behavior. Evidence that primary variant CU traits may indeed be associated with superior performance in at least one language domain is particularly intriguing. It suggests that the atypical language processing emphasized in previous research involving adolescent and adult offenders with psychopathic traits may not be universally detrimental [19, 36, 51].

Youth with high versus low levels of CU traits were found to be comparable in terms of structural language skills, consistent with findings from previous research involving adult offenders [11]. Our data did not support the prediction that higher secondary variant CU traits would be associated with poorer structural language skills. This prediction was based on evidence that youth with such traits are characterized by a number of risk factors that are known to covary with language deficits (e.g., childhood maltreatment, attentional problems, internalizing problems) [6, 28]. It should be noted, however, that this association nonetheless approached significance. This warrants further investigation, as recent research suggests that deficits in structural language skills, specifically, may be associated with reactive aggression [48]. Developing a clear understanding of this association, therefore, has important implications for intervention.

Noteworthy findings were also seen regarding the specific patterns of offending associated with variants of CU traits and oral language skills in our sample. As predicted, those youth offenders with primary variant CU traits and better structural language skills demonstrated significantly higher levels of violent offending than those who had lower structural language skills. Additionally, and consistent with predictions, youth with primary variant CU traits and better pragmatic language skills demonstrated a significantly earlier age of first offence than those with poorer pragmatic language skills. Among youth with low CU traits, those with poorer pragmatic language skills demonstrated a significantly earlier age of first offence than those with better pragmatic language skills. On the whole, this can be seen to reflect the findings of previous data on global CU traits and the much broader construct of verbal ability, from adult offenders [23] as well as youth offenders [43]. This has important implications because both early onset of antisocial behaviour and higher levels of violent behaviour are associated with a more severe criminal career trajectory [13], specifically a life-course-persistent pattern of offending [41]. At the same time, our findings suggest that language-related risk mechanisms for antisocial behavior may play out among distinct subgroups of youth offenders in ways that are more complex than previously thought.

The current findings should be interpreted in light of some limitations. First, this research relates specifically to adolescent males, and it is unknown whether the results generalize to adolescent females. Indeed, research suggests that developmental risk pathways associated with antisocial behavior and CU traits may differ considerably between males and females [47]. Second, although CU traits were indexed using an established measure, they were assessed by youth-self report only. This is noteworthy, as there has been some debate as to whether individuals with CU/psychopathic traits may deliberately under-report their occurrence due to social desirability. Interestingly, measurement research explicitly investigating social desirability and impression management among individuals with psychopathic traits and antisocial behavior has found that self-report measures do not appear to be confounded by ‘faking good’ among such individuals [40, 60]. At the same time, it has been argued that multi-informant measurement may provide the most comprehensive data on CU traits among detained youth [32], and such an approach may further serve to protect against issues such as social desirability. We would therefore recommend that future research incorporates data on these traits from other informants, such as parents and teachers, in addition to youth self-reports. Third, although the Anxious-Depressed subscale used here to operationalize primary and secondary variants of CU traits has been used for this purpose in previous research, some implications of this method should be noted. That is, our findings may not be directly comparable to studies that have used a more pure measure of anxiety for this purpose [8]. Additionally, there is emerging evidence that data on both anxiety and maltreatment may be informative for this grouping [9]. Fourth, the language measures used in this research employed different modalities of assessment and techniques of standardization. While the measures were selected to represent relevant oral language skills, there is ongoing debate regarding the relative merits of different forms of oral language assessment [5]. Finally, although adequate for the planned statistical analyses, the sample size for the current research could be considered relatively small. In future research, a larger sample size would enhance power and facilitate the inclusion of additional variables in analyses.

## Conclusions

The current study is the first, to our knowledge, to examine any language-related variables among youth offenders with distinct variants of CU traits, as well as the first to examine language in conjunction with variants of CU traits and specific patterns of antisocial behavior. Moreover, it is the first to examine associations between specific oral language skills and CU traits variants among children and adolescents of any kind. Reasons for this previous lack of evidence are unclear but may be accounted for by the lack of personality-based research in the fields in which studies of oral language skills have traditionally been conducted (e.g., speech pathology). The finding that for youth offenders with primary variant CU traits, these traits were positively associated with superior pragmatic language skills, is therefore particularly novel. It also has potentially important implications for developmental models of CU traits and antisocial behavior. Pragmatic language has been closely associated with various socio-cognitive skills (e.g., perspective-taking), thereby suggesting that among youth with primary variant CU traits, superior pragmatic language may be implicated in risk processes related to atypical cognitive empathy. This proposition is supported by evidence that primary variant CU traits are positively associated with better cognitive perspective-taking skills among youth offenders [24]. Such evidence is also consistent with accounts of psychopathic individuals whereby they are characterized as proficient in skills for identifying interpersonal vulnerability in others, and effective in manipulating others for self-serving gains [7, 52].

In addition, our findings that primary variant CU traits and specific oral language skills were associated with specific patterns of high-risk offending, provide novel support for the thesis that primary versus secondary variants of CU traits are associated with distinct pathways to antisocial behavior. It is conceivable that the affective deficits associated with CU traits diminish the capacity to succeed in the sort of socially valued activities that would generally be accessible to an individual with superior language skills [23]. An antisocial pathway, including earlier engagement in offending and greater levels of violence, may therefore present as one of the limited options available to individuals with high levels of primary variant CU traits.
